# Persistence of strong and switchable ferroelectricity despite vacancies

**DOI:** 10.1038/srep41301

**Published:** 2017-01-25

**Authors:** Aldo Raeliarijaona, Huaxiang Fu

**Affiliations:** 1 Department of Physics, Applied Physics, and Astronomy, Rensselaer Polytechnic Institute, Troy, New York 12180, USA; 2 Department of Physics, University of Arkansas, Fayetteville, Arkansas 72701, USA

## Abstract

Vacancies play a pivotal role in affecting ferroelectric polarization and switching properties, and there is a possibility that ferroelectricity may be utterly eliminated when defects render the system metallic. However, sufficient quantitative understandings of the subject have been lacking for decades due to the fact that vacancies in ferroelectrics are often charged and polarization in charged systems is not translationally invariant. Here we perform first-principles studies to investigate the influence of vacancies on ferroelectric polarization and polarization switching in prototypical BaTiO_3_ of tetragonal symmetry. We demonstrate using the modern theory of polarization that, in contrast to common wisdom, defective BaTiO_3_ with a large concentration of vacancies 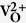 (or 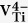, or 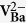) possesses a strong nonzero electric polarization. Breaking of Ti-O bonds is found to have little effect on the magnitude of polarization, which is striking. Furthermore, a previously unrecognized microscopic mechanism, which is particularly important when vacancies are present, is proposed for polarization switching. The mechanism immediately reveals that (i) the switching barrier in the presence of 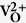 is small with Δ*E* = 8.3 meV per bulk formula cell, and the polarization is thus switchable even when vacancies exist; (ii) The local environment of vacancy is surprisingly insignificant in polarization switching. These results provide profound new knowledge and will stimulate more theoretical and experimental interest on defect physics in FEs.

Vacancies in ferroelectrics (FE), one key class of native defects, are of considerable importance both fundamentally and technologically^1^,^2^,^3^. Fundamentally, vacancies disrupt the interaction among atoms on the microscopic scale, and break the delicate balance^4^,^5^ between long-range and short-range interactions in FEs. Therefore, vacancies can profoundly affect the superior properties that have been found in FEs such as high electromechanical response^6^,^7^,^8^, polarization enhancement in superlattices^9^, large dielectric coefficient^10^,^11^, strong improper ferroelectricity^12^,^13^,^14^,^15^, and unusual phase transition^16^,^17^,^18^. Technologically, vacancies in FEs have been linked to fatigue^19^,^20^,^21^; they also lead to pinning of polarization near domain wall^22^, reduction of polarization magnitude, and increase of coercive field^3^,^23^. Consequently, vacancies hamper the vital applications of FEs in ferroelectric memories and piezoelectric devices^24^,^25^,^26^.

On the other hand, vacancies can be beneficial. In fact, vacancies are pivotal in polarization switching by acting as the nucleation centers of antiphase domains^27^,^28^, as described in the Kolomogorov-Avrami model^29^,^30^ of polarization reversal. In ferroelectric tunnel junctions, the control of polarization switching in terms of nucleation and domain growth is instrumental in the realization of FE memristors^31^. A V_Pb_-V_O_ di-vacancy complex was shown to enhance the local dipole moment in PbTiO_3_^32^. Vacancies were also demonstrated to induce a large electromechanical response in aged BaTiO_3_ crystals^33^ and broaden the dielectric peaks in disordered FEs^34^.

There are reasons that vacancies are detrimental to the polarization in FEs. It is known that Ti 3*d* and O 2*p* hybridization is important for ferroelectric instability^4^,^5^,^35^. The Ti-O chains (or the B-O chains in general) are critical in the formation of ferroelectricity. Creation of Ti or O vacancies disrupts the Ti-O chains, thereby affecting the polarization properties. Furthermore, since polarization switching depends on the local structure and local interaction near defects, existence of vacancies drastically alters the chemical bonding in the neighborhood of the vacancy sites and is thus expected to impact the polarization switching.

However, the profound effects of vacancies (and broken Ti-O chains) on polarization have not been confirmed, and a different conclusion may utterly alter the current knowledge on defect physics in FEs. The lack of confirmation is due to the fact that there are outstanding problems that prevent us from obtaining a microscopic understanding on how vacancies *quantitatively* change the polarization and polarization-related properties. First, the stable state of a vacancy is often charged^36^, and indeed, the most stable O vacancy in PbTiO_3_ is the positively charged 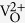. Here we use the notation 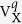 to denote a vacancy of species X carrying an amount of charge *q*. For charged vacancies in a periodic solid, a fundamental question arises, that is, whether polarization is well-defined and physically meaningful. Second, when vacancy is present, there is a possibility that the system could be metallic due to defect states, in which conducting electrons may screen the polarization if there is any. It thus remains unclear whether it is possible to quantitatively determine the magnitude of polarization when vacancies occur in FEs. As a consequence of these standing problems, an accurate understanding of the influence of vacancies, which goes beyond the qualitative argument based on the Ti-O chains, is lacking. This hampers the important effort toward the design of FEs by controlling vacancies.

Another subject of equal importance is the mechanism of polarization switching. Among various models^27^,^28^,^29^,^30^ that describe polarization switching in FEs, a consensus is that the switching is initiated by nucleation of an antiphase domain. However, little is understood on how the antiphase domain forms in the first place. In other words, the knowledge—of how the polarization is reversed *in the neighborhood of the defects* to form an antiphase domain at the key stage of nucleation—is microscopically unknown. In studies of ideal FEs without defects, polarization reversal is realized^1^ by shifting the atoms from one of the degenerate potential wells, say the state with **P** > 0, going through the centrosymmetric configuration, and ending at the enantiomorphic state with **P** < 0. This, to our knowledge, is the only approach employed in studying the energetics of polarization switching. However, when vacancies emerge, the approach may not apply since it does not take vacancies into consideration. Because nucleation often occurs near vacancies, we thus need to account for the presence of vacancies in the process of polarization switching. Specifically we must consider how polarization is switched in the immediate neighborhood of the vacancies, which is in fact the important mechanism for nucleation. Nevertheless, with charged vacancies, it is unclear whether polarization is well defined, and it is even less clear by what mechanism the polarization is switched.

The purpose of this paper is threefold: (i) To show that polarization can be meaningfully defined when charged vacancies occur in FEs, and furthermore can be rigorously calculated, provided that a correct procedure is undertaken. (ii) To demonstrate that, unlike the common wisdom, breaking of the Ti-O chains surprisingly does not significantly reduce the magnitude of polarization. We find that, regardless of the vacancy species (V_Ba_, V_Ti_, or V_O_), the polarization is interestingly similar. (iii) To provide an atomistic mechanism and microscopic insight on how polarization is reversed near vacancies. Using this mechanism we further reveal that the polarization in FEs with vacancies is switchable, with a switching energy barrier comparable to that in ideal bulk. Concurrently, these studies suggest that there are rich and interesting physics to be learnt when vacancy and ferroelectricity coexist.

## Results

### Optimal charge state and the insulating nature

In BaTiO_3_ of *P*4*mm* symmetry, there are two inequivalent O atoms, one directly beneath a Ti atom along the *c*-axis (labeled as apical O1), the other on the base plane of TiO_2_ (labeled as O2). We have computed the vacancy formation and polarization for both V_O1_ and V_O2_ vacancies, and found that they are very similar due to the small *c*/*a* ratio in BaTiO_3_. We will thus present only the results of V_O1_ for apical O1.

**Figure 1:**
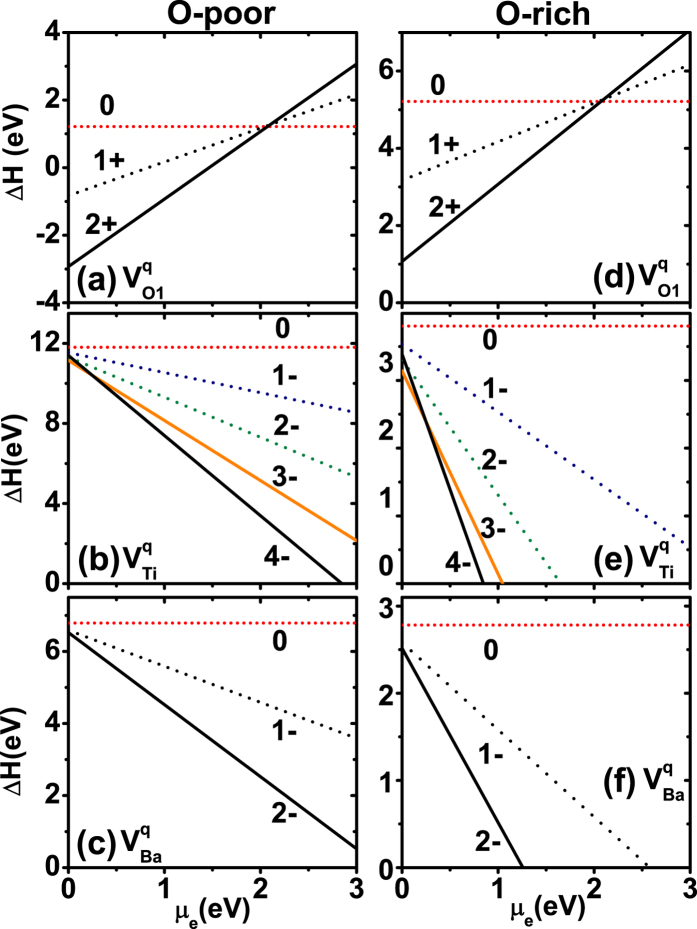
Vacancy formation energy Δ*H* in BaTiO_3_ as a function of the chemical potential *μ*_e_ of the electron reservoir. The left column is under oxygen-poor condition with *μ*_O_ = −4 eV, and the right column is under oxygen-rich condition with *μ*_O_ = 0 eV. In each column, three different vacancy species are considered, namely 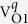, 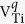, and 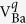. The number labelled near each line is the charge state *q*.

An important question regarding vacancies in FEs concerns which charge state is stable for a given vacancy species. To answer this, we compute, for each vacancy species X, the vacancy formation energy Δ*H* and relative stability of different charge states *q* (see the Methods for detail). All calculations are performed using 3 × 3 × 3 supercells. The obtained formation energies are given in Fig. 1 as a function of the chemical potential, *μ*_e_, of the *electron* reservoir. In experiments, the chemical potential, *μ*_O_, of the *oxygen* reservoir can also be varied, in addition to the fact that *μ*_e_ can be changed. To make our results to be useful for different experimental growth conditions, we consider in Fig. 1 two typical oxygen chemical potentials, where Fig. 1(a–c) correspond to the oxygen-poor condition with *μ*_O_ = −4 eV, while Fig. 1(d–f) correspond to the oxygen-rich condition with *μ*_O_ = 0 eV.

Calculation results in the left column of Fig. 1 reveal that, in most of the *μ*_e_ region when *μ*_e_ is below 2 eV, the most stable charge state with lowest formation energy is 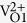 for O vacancy [Fig. 1(a)], 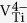 for Ti vacancy [Fig. 1(b)], and 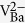 for Ba vacancy [Fig. 1(c)]. This conclusion remains when the growth condition becomes oxygen rich (see the right column of Fig. 1). Figure 1 thus shows that, regardless of whether the system is under the oxygen-poor condition or oxygen-rich condition, *charged* vacancies are energetically more favorable in BaTiO_3_. For each atomic species, the vacancy that is most likely to occur in BaTiO_3_ turns out to be 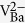, 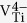, and 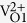, respectively. Figure 1(a) also reveals that, compared to other vacancies, the formation energy of 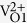 is exceptionally low under the O-poor condition, which will lead to a high concentration of 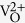. Therefore, under the oxygen-poor condition, 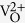 will be dominant.

The most stable charge state with the lowest formation energy (which will be denoted as “the optimal charge state”) is important for the following reason. Since the vacancy concentration 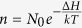 (where *N*_0_ is the density of atomic site and *T* the temperature) decreases exponentially with the formation energy Δ*H*, the optimal charge state with the lowest formation energy thus contributes dominantly to the vacancy concentration for a given atomic species.

Our result that the optimal vacancy state in BaTiO_3_ is charged is consistent with the theoretical findings reported for other oxides. Using different first-principles calculations, the lowest-energy charge state of an O vacancy was found to be 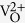 in ZnO (ref. 37) and in SrTiO_3_ (ref. 38), which agrees with the conclusion in the present study. Furthermore, charged vacancies were also observed in experiments, for instance, positively charged C vacancy and negatively charged Si vacancy were detected in SiC using electron paramagnetic resonance^39^,^40^. We thus see that both theories and experiments support the existence of charged vacancies.

We next determine whether the vacancy of the optimal charge state is metallic or insulating. This question seems trivial, but is of important relevance, since defects often introduce partially occupied electron states inside the band gap and make the system metallic^41^, in which the conducting electrons may lead to the screening of ferroelectricity. Although metallic behaviors may occur for some charge states, they by no means occur for all charged states.

**Figure 2:**
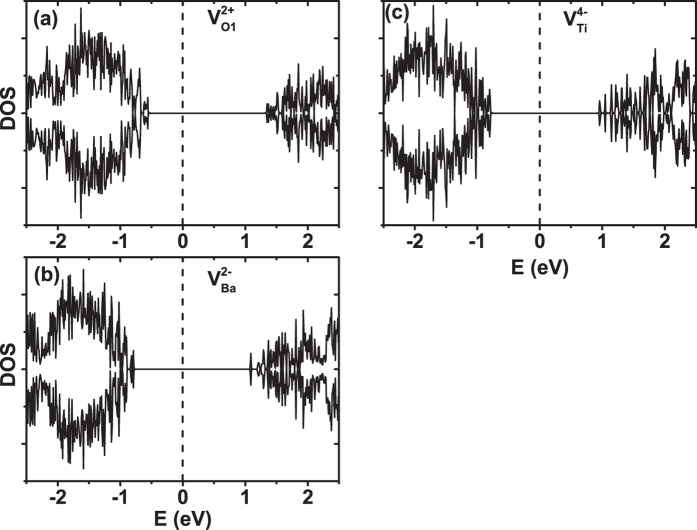
Spin-polarized density of states (DOS) for BaTiO_3_ with (**a**) 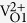, (**b**) 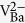, and (**c**) 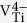 vacancy. The minority DOS component is plotted as negative values. The spin majority and minority DOS are identical, with no ferromagnetism, for 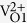, 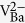, and 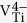 vacancies. The vertical dashed line in (**a**–**c**) marks the Fermi energy.

We calculate the spin-polarized density of states (DOS) for the optimal charged vacancies (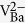, 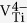, and 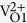), and the result is depicted in Fig. 2. Figure 2(a–c) reveal that a large band gap exists between the fully occupied valence states and the unoccupied conduction states for all three vacancies. BaTiO_3_ with vacancies of optimal charge state is thus found to be an insulator. This conclusion is important, since it tells us that, for these optimal charge states, there are no free electrons available to screen the ferroelectricity provided that ferroelectricity does exist.

### Polarization in BaTiO_3_ with charged vacancy

For charge *neutral* systems, it is known that polarization can be computed by the modern theory of polarization^42^,^43^. However, as determined in previous section, the most stable vacancies 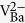, 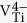, and 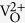 are all charged. A fundamental problem arises when dealing with polarization in a charged system, that is, the polarization itself is not translation invariant and is thus ill-defined. In the following, we will show that even for charged systems, the *change* in polarization is physically meaningful and can be rigorously calculated.

For electrons in periodic solids, it is known that their wave functions are extended and cannot be treated as point charges^44^. Here, we begin by using the centers of Wannier functions^45^ (WanF), and write the polarization in a solid as


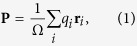


where **r**_*i*_ is either the location of an ion (in which *q*_*i*_ is the point charge of the ion) or the center of a Wannier function (in which *q*_*i*_ is the charge of the Wannier function), and Ω the volume of one cell. If the solid is translated by a constant vector **t** as 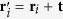, the polarization will then become 
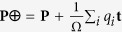
. For charged systems, the second term in **P**′ does not vanish, and the magnitude of polarization is thus not translation invariant.

However, despite that systems are charged, the change in polarization is nevertheless unique. To see this, let the ion or the WanF center change from initial position 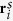 to final position 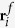 by a displacement **d**_*i*_ as 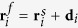 (where **d**_*i*_ is ion dependent, but does not depend on the choice of the vector **t**). Then, although both the initial polarization **P**^*s*^ and the final polarization **P**^*f*^ are not translation invariant as 
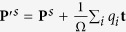
 and 
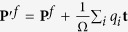
, the change in polarization 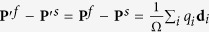 nevertheless does not depend on **t** and is physically meaningful. Therefore the change of polarization is well defined, which coincides with the key requirement in the modern theory of polarization.

The uniquely defined change of polarization for *charged* defects in extended solids is of fundamental significance, since it allows us to compute rigorously the polarization. Furthermore, combined with the insulating nature demonstrated in the previous section, it also establishes that ferroelectricity should exist in BaTiO_3_ with optimally-charged 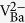, 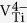, or 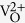 vacancies.

Although charged defect in periodic solids is often neutralized by jellium background, there is one subtle difference between a charged system neutralized with jellium background and a normal charge-neutral system without jellium background, when the electric polarization is concerned. In a normal charge-neutral system, all electrons in the system occupy the Kohn-Sham orbitals, and contribute to the electric polarization, since the Berry phase approach of computing polarization utilizes the occupied Kohn-Sham orbitals to form a determinant^42^,^43^. In contrast, in systems neutralized with jellium background, the jellium charges do not occupy the Kohn-Sham orbitals, and do not contribute to the electric polarization. This is consistent with the fact that the jellium charges are uniformly distributed in space before and after atoms are displaced, thus making no contribution to the change in polarization. Therefore, the polarization change calculated in a system of charged defects comes from the part of the system that excludes jellium, and this part of system is charged.

We next go one step further and determine the magnitude of polarization associated with the optimal charged vacancies, using the modern theory of polarization^42^,^43^. More specifically, for each vacancy species of optimal charge state, polarization change is calculated by adiabatically connecting the centrosymmetric configuration 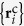 (which has a zero polarization and thus serves as the zero reference) and the LDA-optimized configuration 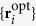, using multiple steps controlled by parameter *λ* (0 ≤ *λ* ≤ 1) according to 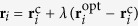. Berry phase calculations are performed at each step so that the polarization change is computed along the connecting path. The polarization value of the configuration at *λ* = 1 (i.e., the polarization of the LDA-optimized atomic structure with vacancies) is what we will focus on. Furthermore, as proved in the Methods section, the computed change in polarization is rigorous and does not depend on whether an atom is fixed in structural optimization.

**Figure 3:**
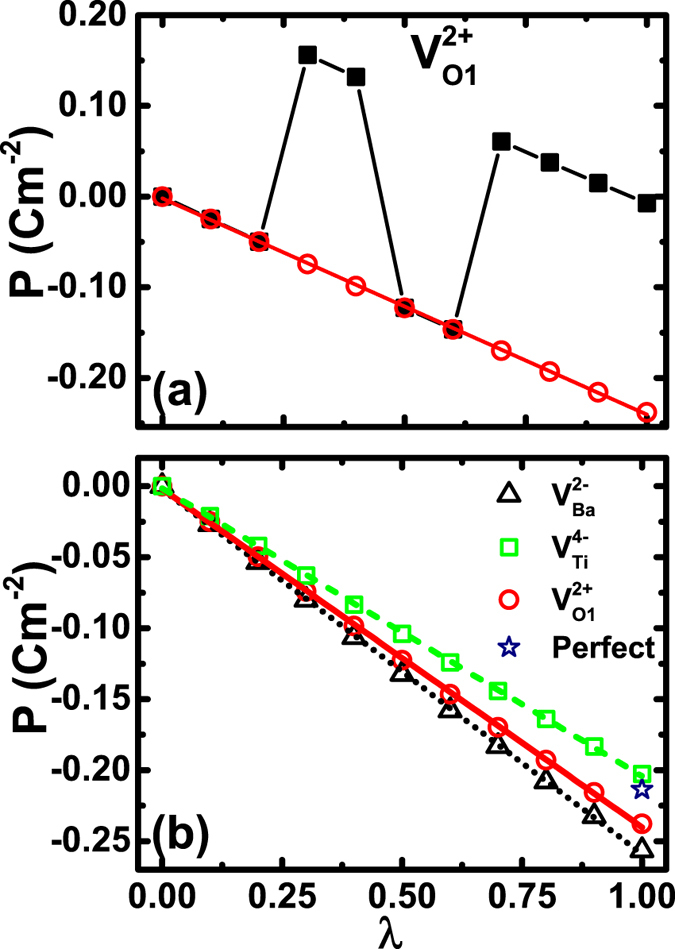
(**a**) Polarization as a function of atomic shifts from the centrosymmetric configuration (*λ* = 0) to the LDA-optimized configuration (*λ* = 1), in BaTiO_3_ with 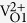 vacancy. Square symbols are the raw data directly obtained from the Berry phase calculations. Empty circles are the result after shifting the raw data by an integer number of **P**_quan_, showing a continuous change of **P** with respect to *λ*. (**b**) Polarization as a function of atomic shifts from the centrosymmetric configuration (*λ* = 0) to the LDA-optimized configuration (*λ* = 1), in BaTiO_3_ with 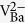, 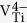, or 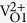 vacancy. The polarizations in (**b**) are obtained by shifting the raw data by an integer number of **P**_quan_. For perfect bulk BaTiO_3_ without vacancy, the first-principles calculated value of polarization is −0.21 C·m^−2^ (i.e., the star symbol at *λ* = 1).

The computed polarizations as a function of *λ*, directly obtained from the Berry-phase calculations, are shown as solid-square symbols in Fig. 3(a) for 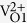. Intriguingly, we see that the calculated polarizations fluctuate widely. We find that this fluctuation is not an artifact, and in fact it originates from the polarization quantum. Calculations of electrical polarization, for systems with vacancies, are often carried out using supercells, which makes the polarization quantum very small. The polarization quantum is defined^42^,^43^ as 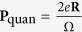, where **R** is the lattice vector along the polarization direction, *e* the electron charge, and Ω the volume of supercell. A large supercell volume leads to a drastic reduction of **P**_quan_. Therefore, when *λ* changes, polarization can easily cross different branches, which gives rise to the polarization fluctuation in Fig. 3(a). The proper value of polarization can be easily determined by shifting the raw data (i.e., the square symbols in Fig. 3a) by an integer number of polarization quanta. After shift, the polarization is depicted as the empty circles in Fig. 3(a). We see in Fig. 3(a) that unlike the fluctuating raw data, the polarization curve of empty circles now is smooth and continuously changing.

Using the same procedure of shifting the raw polarization data by an integer number of **P**_quan_ as in Fig. 3(a), we determine the polarizations as a function of *λ* for 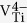 and 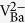 vacancies, and the results are given in Fig. 3(b) in comparison with 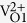. The calculated spontaneous polarization for perfect bulk at *λ* = 1 is also given in Fig. 3(b) for comparison.

Two profound conclusions are revealed in Fig. 3(b). First, the electric polarizations for three vacancies turn out to be nonzero. More specifically, at *λ* = 1, the magnitude of polarization in Fig. 3(b) is 0.24, 0.20, and 0.26 C·m^−2^ for 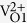, 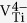, and 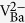, respectively. These values are markedly large. Furthermore, the polarizations for different vacancies are interestingly similar. Note that different vacancies have drastically different environments, for instance, a Ti atom interacts with six oxygen atoms and contributes strongly toward forming the ferroelectricity, whereas a Ba atom plays a much lesser role in developing the ferroelectricity and is largely a spectator^4^,^5^. The close polarizations in Fig. 3(b) show that taking a pivotal Ti atom away from the BaTiO_3_ solid generates similar effect on polarization as taking a Ba atom away, which is indeed striking.

Second, the above polarizations, in BaTiO_3_ with *vacancies*, are comparable to the value *P* = 0.21 C·m^−2^ in a *perfect* bulk without vacancies. Here the polarization in perfect BaTiO_3_ is also obtained from our calculation, which agrees with the experimental value^46^ of *P* = 0.18 C·m^−2^. This second result demonstrates that, unlike the common wisdom in which breaking the Ti-O chains is believed to cause a drastic change to ferroelectricity, our (quantitative) first-principles calculations reveal otherwise, and the electrical polarization is not substantially altered by vacancies of Ti or O. The theoretical vacancy concentration (6 × 10^20^ 1/cm^3^, or one vacancy in 27 bulk cells) in our systems is quite large, and the fact that strong polarization persists in systems of large vacancy concentration is phenomenal.

Our theoretical finding is consistent with available experimental evidence. In experiments, different ferroelectric materials with substantial oxygen deficiency have been synthesized^47^,^48^,^49^, such as Ba_4_Nd_2_Fe_2_Nb_8_O_30_ (ref. 47), Bi_3.25_La_0.75_Ti_3_O_12_ (ref. 48), as well as BaTiO_3_ (ref. 49). Despite the existence of oxygen vacancies, significant FE spontaneous polarizations have been observed in polarization-vs-electric-field curves^47^,^48^,^49^. These experiments provide strong support to our theoretical result that ferroelectricity can indeed exist when vacancies are present.

The unsuppressed polarization, despite the existence of vacancies, can be explained as follows. By examining atomic positions, we find that, for atoms far away from the vacancy, the Ti-O2 buckling distance in BaTiO_3_ with vacancies is comparable to the value in perfect bulk. For instance, in 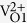, the Ti-O2 buckling distance far away from the vacancy is ~0.09 Å, which is similar to the value of 0.08 Å in perfect bulk BaTiO_3_. This indicates that the effect of vacancy is effectively screened due to the large static dielectric constant of FE, which reduces the local-environment effect of the vacancy and maintains the polarization. Furthermore, as will become clear below, the average effective charges in BaTiO_3_ with vacancies are comparable to the values in perfect bulk, indicating to some extent that the long-range interaction is not significantly altered, which helps to preserve ferroelectricity.

To explain why the polarization in Fig. 3(b) is the largest for 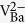 and smallest for 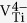, we have performed linear response calculations of large supercells (with 134 atoms) to determine the effective charges *Z*^∗^. We will present the 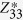 values, which are related to the *c*-axis polarization in tetragonal structure. The average 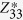 values of Ba, Ti, O1, and O2 atoms are respectively 2.83, 6.36, −5.11, −2.02 in 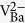; 2.85, 6.31, −5.09, −2.11 in 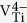; 2.83, 6.17, −5.08, −2.02 in 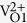. We have also calculated the 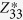 values in perfect bulk, which are 2.81, 6.53, −5.05, −2.10 for Ba, Ti, O1, and O2 atoms, respectively. We did not find a strong correlation between the effective charges and the polarization in Fig. 3(b). Furthermore, we have determined the atomic displacements with respect to the centrosymmetric configuration for each vacancy. In order to compare the magnitudes of displacements in different vacancies, we put the *average* displacement of the Ba sublattice to be zero in each vacancy. The average *c*-axis atomic displacements (in Å) of Ba, Ti, and O atoms are 0.0, 0.051, −0.060 in 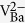; 0.0, 0.046, −0.055 in 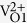; 0.0, 0.040, −0.049 in 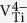. We see that the relative Ti-O displacement is the largest in 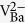, the second largest in 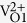, and the least in 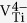. This correlates well with, and may thus explain, the magnitude of polarization in Fig. 3(b), where the magnitude of polarization decreases in the order of 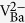, 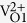, and 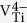.

It is surprising that the polarization of defective system such as in 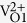 and in 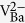 could be larger than that in perfect bulk [see Fig. 3(b)]. This may be explained again in terms of the average Ti-O relative displacement. The average Ti-O relative displacement is 0.111 Å in 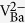 and 0.101 Å in 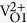, both of which are slightly larger than the value (0.090 Å) in perfect bulk.

We would also like to be cautious and discuss the effects of other charge states. First, there is possibility that other charge states than the optimal one may occur, e.g., 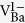, 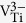, or 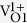. Since the non-optimal charge states possess a higher formation energy, and since the defect concentration declines exponentially with the formation energy, the concentrations of the non-optimal charge states are anticipated to be significantly less and thus play only a minor role. Second, we recognize that non-optimal charge states could introduce extra electrons or holes that may partially screen the ferroelectricity. The extra charge carriers may either be trapped at localized defect states, or be able to move freely. If these charge carriers are trapped, conductance occurs often by the slow hopping process. As a result, the screening caused by the trapped electrons or holes will be weak and incomplete, in which ferroelectricity is likely to remain. On the other hand, if the extra charge carriers are free and mobile, they will move out of the FE solid when the sample is connected to electrodes during the polarization measurement, leaving the localized charges in the sample which can only partially screen the ferroelectricity. Again, ferroelectricity likely remains.

### Polarization switching mechanism in FEs with vacancies

While polarization persists in BaTiO_3_ with vacancies, it remains an intriguing question whether this polarization can be switched. Since vacancies drastically distort the local structure near the defects, the polarization may, or may not, be switchable, and a non-switchable polarization will not be technologically useful. To address this critical issue, we begin by describing an unusual polarization-switching phenomenon that occurs when vacancies are present in FEs. It is well known that, for *perfect* bulk perovskites without vacancies, the B-site atoms need to pass the centrosymmetric positions in order to reverse the polarization^1^,^4^, which is termed as the centrosymmetric switching pathway.

**Figure 4:**
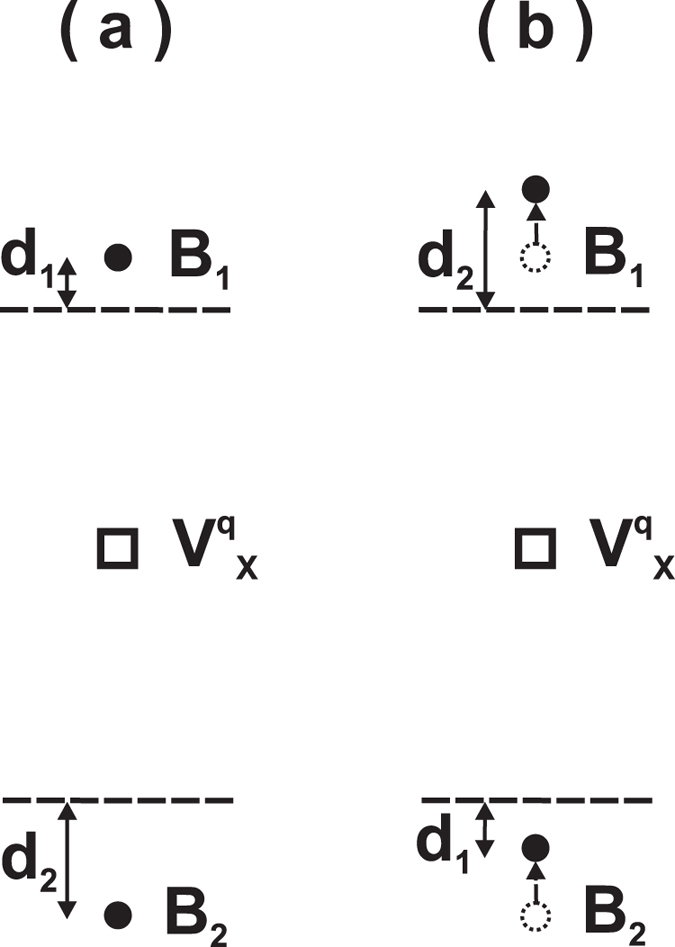
Polarization-switching pathway in the presence of a 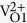 vacancy: (**a**) the initial configuration with an asymmetric structure, where B_1_ and B_2_ are B-site atoms, the dash lines indicate the centrosymmetric planes of the bulk 5-atoms cell for these two atoms, and 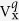 is the vacancy. Polarization is along the B_1_-B_2_ direction. (**b**) Polarization switching in which B-site atoms do not cross the centrosymmetric positions. Empty circles indicate initial positions and solid circles the final positions.

Interestingly, when vacancies exist in FEs, we find that the B-site atoms in the vicinity of the vacancy need *not* pass the centrosymmetric positions, but can nevertheless switch the polarization. This finding is of important consequence, since the centrosymmetric position often possesses a large energy barrier, and bypassing this position can lead to a significant reduction in the polarization-switching barrier (see below). Using the optimized atomic positions obtained from our first-principles calculations, we illustrate in Fig. 4(a) the locations of two B-site atoms next to an oxygen 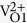 vacancy, where only B-site atoms (B_1_ and B_2_) and vacancy 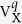 are shown for the sake of clarity. The centrosymmetric plane of each B_*i*_ atom in its bulk unit cell is indicated by the dash line in Fig. 4(a), and *d*_*i*_ is the *z*-direction distance between atom B_*i*_ and the centrosymmetric plane. We emphasize in Fig. 4(a) that, in the vicinity of the vacancy, B_1_ and B_2_ are not both above (or both below) the centrosymmetric planes. Instead, B_1_ is above, but B_2_ is below, its centrosymmetric plane because the strong Coulomb interaction of the 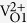 vacancy repels the two B-site atoms. Meanwhile, atoms B_1_ and B_2_ in Fig. 4(a) are not mirror symmetric from the vacancy 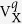 (namely *d*_1_ ≠ *d*_2_), due to the existence of ferroelectricity. Polarization in Fig. 4(a) is along the B_1_-B_2_ direction.

In the neighborhood *near* the vacancy, the correct polarization-switching pathway is described in Fig. 4(b). Our first-principles calculations confirm that with this path the polarization is indeed switched. By symmetry, it is easy to understand that, after switching, the reversed configuration with an opposite polarization should be the mirror-reflected configuration of Fig. 4(a), in which the mirror plane is located at the vacancy site and is perpendicular to the polarization direction. Therefore, during the polarization switching, B_1_ in Fig. 4(b) moves from the (initial) dotted-circle position to the (final) solid-circle position, i.e., B_1_ moves toward the mirror-reflected position of B_2_. With respect to perfect bulk, one key difference for polarization switching in FEs with vacancies is that, in Fig. 4(b), B_1_ does not cross the centrosymmetric plane. Similarly B_2_ does not cross the centrosymmetric plane either, and it moves toward the mirror-reflected position of B_1_. Since each atom shifts toward the mirror-reflected position of its corresponding atom, the polarization switching is guaranteed.

The above proposed polarization-switching mechanism also correctly reproduces the limit of the bulk model when we consider the spatial region *far away* from the vacancy. Far away from the vacancy, the electrostatic Coulomb interaction caused by the charged defect drastically decreases, and the vacancy-induced atomic relaxations become weaker than the ferroelectricity-induced off-center displacements. As a result, the atomic arrangement far away from the vacancy is similar to that in perfect bulk FE, where the B_1_ and B_2_ atoms will be both below, or both above, the centrosymmetric dash planes due to the existence of ferroelectricity (which is unlike Fig. 4a). The requirement that B_1_ moves toward the mirror-reflected position of B_2_ during polarization switching then leads to the fact that B_1_ must cross the centrosymmetric plane, which resembles, as it should, the polarization reversal pathway in perfect bulk.

**Figure 5:**
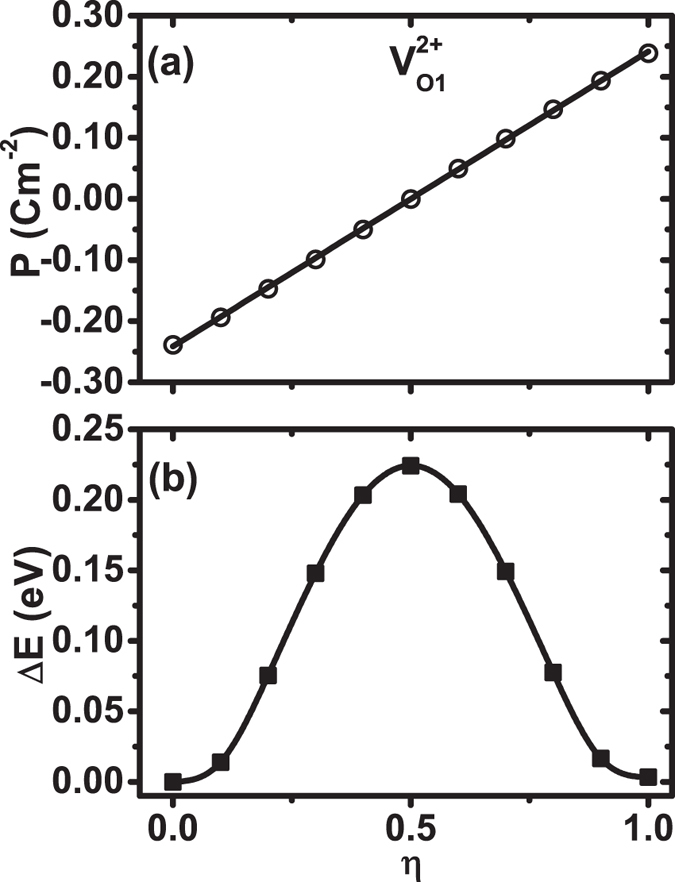
(**a**) Polarization as a function of parameter *η* during the switching for BaTiO_3_ with 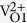 vacancy. *η* = 0 is the initial configuration before switching; *η* = 1 is the final configuration after switching. (**b**) Change in energy Δ*E*(*η*) = *E*(*η*) − *E*(*η* = 0) as a function of *η* during polarization switching for BaTiO_3_ with 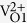 vacancy, using the proposed switching pathway in Fig. 4(b).

To demonstrate that the proposed path in Fig. 4(b) can indeed switch the polarization, we perform polarization-reversal calculations for BaTiO_3_ with 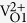 vacancy. More specifically, we calculate the Berry-phase polarizations for a series of intermediate configurations according to 
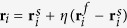
, where 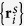 are the atomic positions of the starting configuration [Fig. 4(a)] and 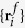 are the atomic positions of the final (reversed) configuration [Fig. 4(b)]. *η* is the parameter specifying an intermediate configuration during the switching (0 ≤ *η* ≤ 1). The computed polarizations, after shifting by an integer number of polarization quanta, are shown in Fig. 5(a). We see in Fig. 5(a) that the polarization at the final (*η* = 1) configuration is +0.24 C·m^−2^, which is exactly opposite to the polarization value of −0.24 C·m^−2^ at the initial (*η* = 0) configuration. This convincingly demonstrates that, when vacancies are present, the pathway proposed in Fig. 4(b) indeed switches the polarization.

### Polarization-switching barriers

We have calculated for 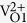 the energy barrier of polarization switching, shown in Fig. 5(b). Since the ferroelectric state considered here is a single domain polarized over the entire supercell, the polarization-switching barrier is thus the energy needed to switch the polarization of the supercell. Figure 5(b) reveals important new knowledge regarding the influence of vacancies. It tells that the energy barrier is 0.224 eV per supercell, consisting of 27 bulk cells. Converted into the energy barrier per bulk cell, it amounts to an energy barrier of 8.3 meV per bulk cell, which is small compared to the barrier height^4^ of ~200 meV per bulk cell in PbTiO_3_. We also compute the switching barrier for perfect bulk BaTiO_3_ without vacancy, and the barrier is 4.0 meV per bulk cell. We thus find that the switching barrier in BaTiO_3_ with 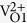 vacancies is on the same order of magnitude as in a perfect bulk. By examining atomic positions, we further find that, at *η* = 0.5 in Fig. 5(b), atoms near the vacancy are half way moving from the initial positions [i.e., the empty circles in Fig. 4(b)] to the final positions [i.e., the solid circles in Fig. 4(b)], and meanwhile, atoms further away from the vacancy are at their respective centro-symmetric planes (thus forming the switching barrier). The reversed polarization in Fig. 5(a) and the small energy barrier in Fig. 5(b) demonstrate that, despite the presence of vacancies, the electrical polarization in BaTiO_3_ is switchable.

**Figure 6:**
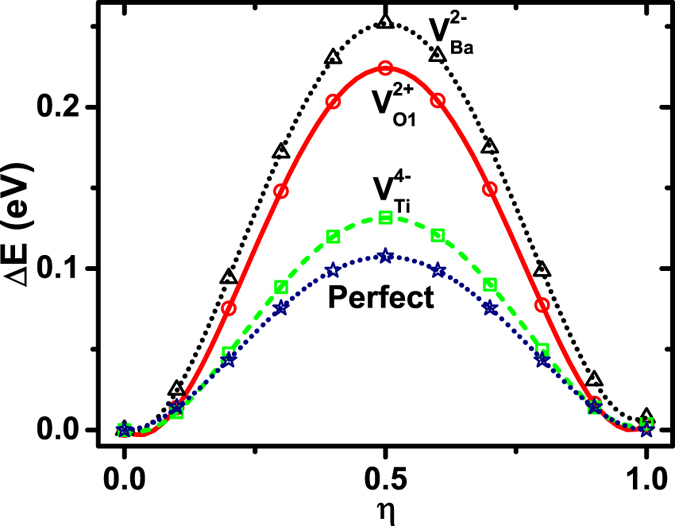
Polarization-switching energy barriers for 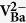 (triangle symbols), 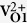 (circle symbols), and 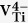 (square symbols). The switching barrier for a 3 × 3 × 3 supercell of a perfect bulk is also given for comparison (star symbols).

Furthermore, it is interesting to compare the switching barriers for different vacancies, using the proposed non-centrosymmetric pathway. The results are given in Fig. 6, in comparison with the switching barrier of a 3 × 3 × 3 supercell in perfect bulk. Although 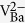, 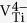 and 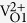 exhibit very different local environments, Fig. 6 shows that the polarization-switching barriers are nevertheless similar. The barriers are found to be 0.252, 0.132, 0.224 eV per supercell—or equivalently, 9.3, 4.9, 8.3 meV per bulk cell—for 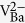, 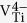 and 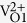, respectively. Our first-principles calculations thus reveal that polarization can be reversed for all three vacancies (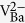, 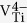, and 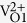).

A correlation exists between the switching barrier in Fig. 6 and the (average) relative Ti-O displacement. As described previously, the relative Ti-O displacements in three vacancies decrease in the order of 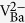, 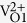, and 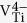, which correlates with the barrier heights in Fig. 6. The smallest relative Ti-O displacement in 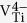 indicates that, compared to 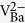 and 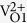, the LDA-optimized non-centrosymmetric configuration in 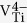 is closer to the centrosymmetric configuration, which may lead to a smaller energy difference (i.e., a smaller switching barrier) between the above two configurations.

We like to take extra caution and evaluate the magnitude of correction to be caused by the use of finite-size supercell in our calculations. Using the average dielectric constant 
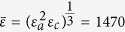
 (which is obtained from the dielectric constants *ε*_*a*_ = 4000 and *ε*_*c*_ = 200 that are within the typical experimental ranges of dielectric constants^1^ in tetragonal BaTiO_3_) and the formula of supercell image charge^50^, we estimate that the finite-size supercell correction to the formation energy of 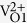 is about 1.6 meV per supercell. This is much smaller than the switching barrier of 224 meV for 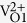. The small correction is attributed to the large static dielectric constant in BaTiO_3_. Therefore the finite-size correction will not alter our conclusion. We also recognize that the dielectric constant is highly anistropic in BaTiO_3_ and the Makov-Payne correction may not work well. To estimate the error of isotropic approximation, we compute the correction using the low limit of dielectric constant, i.e., *ε* = *ε*_*c*_, and the calculated finite-size supercell correction is 12 meV, which is still much smaller than the switching barrier of 224 meV for 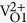. Furthermore, since the correction is of similar magnitude for different intermediate configurations during switching, the error caused by such correction will be largely offset.

Our theoretical finding of switchable polarization and low switching barrier is able to explain the interesting experimental observations that have been reported^47^,^48^,^49^. By measuring hysteresis in different FE materials with oxygen vacancies (which include Ba_4_Nd_2_Fe_2_Nb_8_O_30_ in ref. 47, Bi_3.25_La_0.75_Ti_3_O_12_ in ref. 48, and BaTiO_3_ in ref. 49), polarization switching was clearly observed as the polarization reverses its direction in hysteresis loop. These measurements show evidence that polarization in FEs with vacancies is indeed switchable, consistent with our theoretical result. Furthermore, we recognize that domain walls, grain boundaries, or charge-compensating defects could be very different in different materials. The fact that polarization exists and is switchable in different FE materials with vacancies^47^,^48^,^49^ implies that the conclusion is generally applicable.

Since the topic of this paper is limited to the kinetics of intrinsic polarization switching, the dynamics of the switching process and the pinning of domain walls are beyond our approach. As a consequence of the limitation of our approach, the measure (which we use to judge whether polarization can be switched) is largely based on the comparison with the perfect bulk. Experiments showed that polarization in perfect BaTiO_3_ bulk is switchable^1^. Using the Ginzburg-Landau theory, it can be shown (by following a similar approach as in ref. 51) that the intrinsic coercive field *E*_*c*_ is proportional to 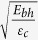, where *E*_*bh*_ is the barrier height and *ε*_*c*_ is the dielectric constant along the polarization direction. Since *E*_*bh*_ in 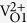 is about 2.1 times of the value in perfect BaTiO_3_, *E*_*c*_ in 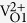 will be about 1.45 times of bulk value, which is not prohibitively large. Basing on this estimation, we thus think that the polarization in BaTiO_3_ with vacancy is likely to be also switchable. Indeed, as described above, it was found in experiments that polarization in different FE materials with oxygen vacancies is switchable^47^,^48^,^49^, which to some extent supports our estimate. In PbTiO_3_, the extrinsic effect such as low domain wall energy may also contribute to the switching of polarization, in addition to intrinsic *E*_*bh*_ and *ε*_*c*_.

Meanwhile, we point out that our results of barrier heights are obtained under zero external field. Determination of barrier heights under *finite* electric fields requires the structural optimization and density functional calculations under finite electric fields^52^. However, these calculations are time-consuming for large supercell of 135 atoms, and we will leave them to a future study. Nevertheless, a qualitative picture may help to address the question. Since vacancy of optimal charge state is an insulator, the defective system with vacancies of optimal charge state will respond to applied electric field in a similar manner as perfect BaTiO_3_ (which is also an insulator). More specifically, under *weak* electric field, the barrier height will be similar to that under zero field, according to the perturbation theory. Under finite electric field which is collinear to the polarization, one ferroelectric potential well will rise in energy (thus reducing the barrier height) while the other well will decrease in energy, which will eventually lead to polarization reversal. If the finite electric field is canted (i.e., non-collinear) with polarization, polarization rotation may occur^7^.

The discovery that the polarization-switching barriers are comparable for different vacancy species is intriguing. Since different vacancies have very different local environments, the finding suggests that the local environments of different vacancies play only a minor role in polarization switching. We now provide a microscopic origin responsible for this remarkable result. For this purpose, we study the mechanical work that is needed to move atoms during the polarization switching. More specifically, we calculate, for each considered vacancy species, the adiabatic work *W* = −∑_*i*_∫**F**_*i*_ · *d***r**_i_ performed to displace the atoms (not vacancies) from the initial configuration *η* = 0 to the configuration of *η* = 0.2, where **F**_*i*_ is the force on atom *i*. We carry out the above integral in *W* approximately using the Simpson’s three-point integral formula. This approximation works better from *η* = 0 to *η* = 0.2, since forces **F**_*i*_ in this *η* region change rather smoothly near the *η* = 0 equilibrium. The mechanical work *W* is an intuitive estimation of the switching barrier.

To uncover the contribution of the local environment, we further separate the atoms in the summation ∑_*i*_ of work *W* into two groups: the first group (termed as group I) includes those atoms that are located within a chosen cutoff radius (*r*_cut_) from the vacancy, and the second group (termed as group II) includes those atoms that are outside the cutoff radius. The *r*_cut_ value in our calculations is 0.80*a*_0_ for 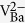, 0.60*a*_0_ for 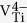, and 0.58*a*_0_ for 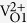, where *a*_0_ is the bulk inplane lattice constant. We choose the above *r*_cut_ values because we find that, after structural optimization, atoms immediately near the vacancy have largest relaxations, and their atomic positions substantially differ from those in perfect bulk. For instance, for 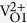, the nearest two Ti atoms have the largest relaxations and are most affected by the existence of vacancy. We thus choose *r*_cut_ = 0.58*a*_0_ for 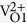 so that these two Ti atoms are in group I. By doing this, contribution of the atoms in group I to the mechanical work thus largely reflects the influence of local environment on switching.

**Figure 7:**
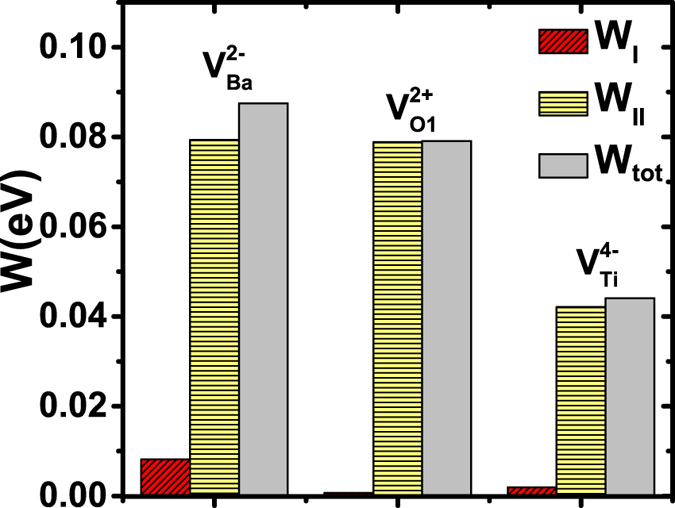
Mechanical works to move atoms in group I (W_I_) and in group II (W_II_), and total work (W_tot_), during the polarization switching process between the initial configuration (*η* = 0) and the configuration of *η* = 0.2, for different vacancies. For 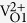, the contribution W_I_ is too small to be seen in the graph. Note that the mechanical work in this figure is the work to move atoms (not vacancies).

Figure 7 depicts the mechanical works to move atoms in groups I and II. We see that, for each vacancy, nearly 90% of the total mechanical work is used to move atoms in group II. In sharp contrast, the work W_I_ performed to move atoms in group I is small, which reveals that the local environment near vacancy indeed is insignificant in the polarization switching. This can be explained by the fact that the atoms near vacancy (i.e., atoms in group I) do not cross the centrosymmetric plane—a plane possessing a large energy barrier, thereby giving rise to only a small contribution to the switching barrier.

We also examine the work per atom of the same species in group I and group II, and find that the work per atom is also small in group I. With the above choice of *r*_cut_, there is only one atomic species in group I for each vacancy. For example, for 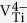, there are six O atoms in group I (i.e., oxygen is the only species in group I), and we thus compare the work per O atom in group I and the work per O atom in group II. We find for 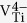 that the ratio (*R*) between the work per O atom in group II and the work per O atom in group I is 6.0, showing that the work per atom is considerably larger in group II. This again demonstrates that atoms in group I play only a minor role in contributing to the switching barrier. Similar conclusion is also true for 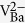 and for 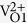. Furthermore, the conclusion does not depend significantly on which *η* region we consider. We have calculated the mechanical works from *η* = 0 to *η* = 0.5, in contrast to the mechanical work from *η* = 0 to *η* = 0.2. We find that the ratio *R* changes only slightly by less than 15%. For example, for 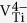, the ratio is 5.1 from *η* = 0 to *η* = 0.5, which is comparable to the ratio of 6.0 from *η* = 0 to *η* = 0.2. In both regions of *η*, atoms in group I play a minor role.

Since the work contribution from atoms in group II dominates and atoms in group II are more bulk-like, one may wonder why the switching barrier in BaTiO_3_ with vacancy (e.g., 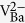) is significantly higher than that in perfect bulk. This can be explained by the fact that atoms in group II in defective BaTiO_3_ (particularly those atoms in the intermediate distance from the vacancy) contribute to the switching barrier differently than the same type of atoms in perfect bulk, due to the combined effect of the residual electrostatic Coulomb interaction (which is caused by charged vacancy and is not fully screened) and the short-range atomic relaxation (which is caused by the absence of an atom) in defective system. Indeed, for 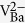 whose switching barrier is significantly higher than that in perfect bulk, we numerically find that, from *η* = 0 to *η* = 0.2, the average contribution per Ti atom in group II to the total work is 1.57 meV in 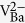, which is substantially larger than the average contribution per Ti atom in perfect bulk (0.65 meV).

Although we consider only BaTiO_3_, we nevertheless investigate three different vacancies (V_Ba_, V_Ti_, and V_O_). Our conclusions—namely that polarization is unsuppressed by vacancies, and is switchable with low switching barriers—apply to different vacancy species of optimal charge states, and are thus rather general. Furthermore, in different FE compounds such as PbTiO_3_ and BaTiO_3_, the Ti-O bonds are similar, and it is thus reasonable that the conclusions may apply to other titanate compounds. Here, it may also be useful to point out that the screening of vacancy charge and its effect on polarization switching are largely determined by the component of dielectric constant along the *c*-axis polarization direction (not by the dielectric components along the lateral *ab* directions), since switching involves mainly the structural change along the *c*-axis. In tetragonal BaTiO_3_, although the dielectric constant is very high along the *ab* plane, the dielectric constant *ε*_*c*_ along the *c*-axis is about 200. Since the *ε*_*c*_ dielectric constants in BaTiO_3_, PbTiO_3_, and CaTiO_3_ are on the similar order of ~100 (*ε*_*c*_ of SrTiO_3_ is even larger), the screening effect will thus be similarly effective in these perovskite solids, as compared to in semiconductors (where *ε* is on the order of 10). We thus anticipate that a similar conclusion can be drawn for other ferroelectric or incipient perovskites.

Our study also has important implication for polarization imprinting. It is known from experiments^1^,^2^,^3^ that polarization often becomes harder to switch after a great number of switching cycles, a phenomenon called imprinting. Since isolated vacancies do not considerably change the switching barrier as demonstrated in this study, the imprinting then must have originated from the *extended* defects (e.g., defect lines, defect clusters, etc.) that develop after individual defects migrate to form an aggregate. Here it may be useful to contrast our results with those of ref. 22, where neutral oxygen vacancies were shown to have considerable effects by pinning domain walls. In ref. 22, a supercell of 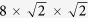 was used, in which the two lateral cell lengths are small and the vacancy-vacancy interaction could be strong along the lateral directions. Also, due to the small lateral cell lengths, the atoms between two neighboring vacancies could be substantially affected, which may lead to large vacancy-induced effects. The defect simulated in ref. 22 is more like an extended planar defect rather than a point defect. In contrast, the size of supercell in this study is 3 × 3 × 3, in which vacancy is more like a point defect and the vacancy-vacancy interaction is small along all three directions. The current study shows that isolated point vacancies of optimal charge states have an interestingly small effect on switchable polarization, although the defect concentration is high (one vacancy per 27 bulk cells). In fact, combining the results in ref. 22 and in the current study supports to some extent the above speculation that extended defects, which form after individual vacancies migrate, could be responsible for imprinting.

## Discussion

Vacancies in FE materials are of critical interest in studying ferroelectric properties and polarization switching. However, quantitative *ab initio* understandings have been elusive and profoundly difficult for decades. In this study, we established two key results of fundamental relevance: (i) In FEs with charged defects, the change in polarization can be meaningfully calculated; (ii) Strong ferroelectricity was demonstrated to persist in BaTiO_3_, despite the presence of vacancies 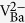, 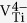, or 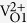 of optimal charge state.

Furthermore, using density-functional calculations and the modern theory of polarization, we revealed that polarization in BaTiO_3_ is interestingly similar for different vacancy species. The results also challenge the conventional wisdom in FEs, showing that ferroelectricity can survive the breaking of Ti-O bonds.

Moreover, we provided a microscopic polarization-switching mechanism, which yields (at atomic scale) important knowledge on the polarization reversal when vacancies emerge in FEs. In BaTiO_3_ with 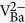, 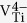, or 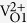, polarization was found to be switchable, with a low switching barrier less than 10 meV per bulk cell. The B-site atoms in the neighborhood of vacancy need not cross the centrosymmetric plane during polarization switching, which avoids the energy maximum located at the local centrosymmetry plane and thus gives rise to a reduced switching barrier. The mechanism further leads to another new discovery that the local environment near vacancy plays only a minor role in the polarization switching. It is thus possible to design defects with complex local structures that nevertheless possess a switchable polarization of a low switching barrier.

Considering the fundamental and technological importance of native vacancies in FEs, and considering that a quantitative understanding of their profound effects is still scarce, we hope that the unusual physics revealed in the current study will stimulate more theoretical and experimental interest in the field of defect physics in FEs.

## Methods

### Total energies and forces

We perform first-principles calculations of total energy, force, and structure optimization using the density functional theory (DFT) within the spin-polarized local density approximation^53^ (LDA) to investigate the vacancy properties in tetragonal BaTiO_3_ of *P*4*mm* symmetry, a phase stable near room temperature. Spin-polarized calculations are performed since charged vacancies may introduce magnetism. Norm-conserving pseudopotentials^54^ are used to describe the interaction between core and valence electrons. Ti 3*s* and 3*p* semi-core orbitals are treated as valence states; quality of our pseudopotentials is good^55^. The energy cutoff for plane-wave expansion of wavefunction is 90 Ry, which is sufficient. All calculations are performed using the Quantum-espresso package^56^,^57^. For perfect bulk BaTiO_3_ of *P*4*mm* symmetry, our calculated lattice constant *a* = 3.93 Å and tetragonality *c*/*a* = 1.007 are in good agreement with other calculations (for example, *a* = 3.945 Å and *c*/*a* = 1.009 in ref. 58).

For BaTiO_3_ with vacancies, we use 3 × 3 × 3 supercells of 135 atoms in order to reduce the interaction between vacancies. A vacancy is placed at the center of each supercell. Three vacancy species (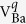, 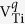, and 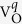), each with different charge states *q*, are considered. For every vacancy species and every charge state, atomic positions are fully relaxed before other properties are calculated. A 4 × 4 × 4 **k**-mesh is used in the calculations, which is sufficient for a large supercell consisting of 27 bulk cells.

Charged vacancies have been firmly observed in experiments using electron paramagnetic resonance^39^,^40^ (EPR) or positron annihilation spectroscopy^59^ (PAS). While vacancies are charged, the whole crystal in experiment is often charge neutral, with compensating charges at surface, grain boundaries, or localized trapping centers. These localized compensating charges often do not actively participate in changing the properties of the interior bulk, while they provide a charge background to neutralize the system. To mimic this situation, jellium charges are used in our DFT calculations to avoid the Coulomb divergence in periodic systems, which is a standard method commonly employed in the studies of charged defects^36^,^60^,^61^.

### Vacancy formation energy

For a given vacancy species, the stability of different charge states is determined by the vacancy formation energy Δ*H*, calculated using the chemical-potential approach^36^,^60^,^62^ as


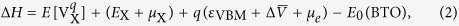


where 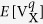 is the total energy per supercell for BaTiO_3_ with vacancy 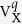, *E*_X_ the total energy per atom in an elemental solid of species X (for oxygen, *E*_X_ is the energy per atom in an O_2_ molecule), *μ*_X_ the chemical potential of the atomic reservoir of species X, *ε*_V*BM*_ the Kohn-Sham orbital energy of the valence band maximum (VBM) in a perfect BaTiO_3_ crystal, 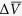 the difference in the average potential between a perfect crystal and a crystal with vacancy, *μ*_*e*_ the chemical potential of the electron reservoir, and *E*_0_(BTO) the total energy of perfect BaTiO_3_. The descriptions on how to compute each term in Eq. (2) were given in ref. 62 for PbTiO_3_, and will not be repeated here.

The chemical potentials (*μ*_X_) of different atomic reservoirs are constrained using the thermodynamical conditions and the requirement that there is no appearance of unwanted secondary phases. The method was described in ref. 63. Furthermore, since in experiments the chemical potential *μ*_O_ of the oxygen reservoir is often used to control the growth, we simulate this situation by determining the vacancy formation energy under different chemical potentials of *μ*_O_. From the thermodynamical constraints, we find for BaTiO_3_ that *μ*_O_ needs to satisfy −5.7 ≤ *μ*_O_ ≤ 0 eV. The maximally allowed *μ*_O_ = 0 eV corresponds to the O-rich condition, and the minimally allowed *μ*_O_ = −5.7 eV corresponds to the O-poor condition. For a given *μ*_O_, our calculations yield that *μ*_Ba_ and *μ*_Ti_ must satisfy −7.39 ≤ *μ*_Ba_ + *μ*_O_ ≤ −5.70 eV and −12.13 ≤ *μ*_Ti_ + 2*μ*_O_ ≤ −10.44 eV. We choose the middle point of each allowed range, namely *μ*_Ba_ + *μ*_O_ = −6.545 eV and *μ*_Ti_ + 2*μ*_O_ = −11.285 eV, to determine *μ*_Ba_ and *μ*_Ti_. The above chemical potentials of atomic reservoirs differ slightly from those in ref. 63 because of different computational methods.

### Computation of polarization

Total electric polarization includes the ionic (**P**_ion_) and electronic (**P**_el_) contributions. Computing **P**_ion_ is straightforward using point charges. **P**_el_ is calculated using the sophisticated modern theory of polarization^42^,^43^ according to 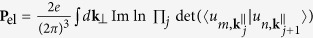, which is related to the geometrical phase of the determinant formed by the occupied Bloch states *u*_*n***k**_. **P**_el_ can be further analyzed using the theory of polarization structure^64^.

In the modern theory of polarization, only the polarization change is physically meaningful. Here, we calculate the polarization change Δ*P* between the LDA-optimized atomic configuration and the centrosymmetric configuration; the latter configuration with zero polarization serves as the reference for computing polarization. More specifically, we use the following procedure to compute the polarization for BaTiO_3_ with vacancy. First, for a given charge state of a given vacancy, we perform LDA calculations to determine the optimized atomic geometry (denoted as 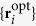). Then we construct a centrosymmetric configuration 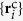 with zero polarization, by using the same cell parameters as the LDA-optimized structure but placing atoms in their high-symmetry positions. We next use multiple intermediate steps to connect the centrosymmetric configuration and the LDA-optimized configuration, according to 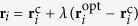 with 0 ≤ *λ* ≤ 1. Berry phase calculations are performed at each step so that the polarization change is computed along the connecting path. The polarization value of the configuration at *λ* = 1 is what we need.

**Figure 8:**
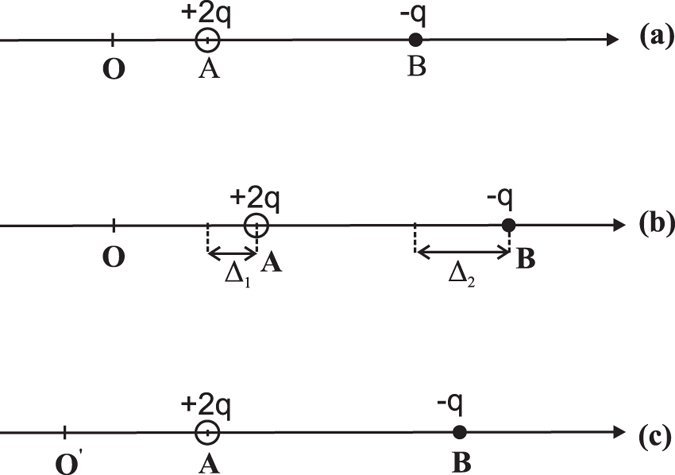
Schematic illustration of displacements of two point charges in the determination of polarization. (**a**) Initial positions of charges A and B, and the origin O of the coordinate system; (**b**) Final positions of A and B after they are displaced; (**c**) Shifting the charges in (**b**) so that the position of A is fixed at the same location as the initial position in (**a**).

One can show in a straightforward manner that for charged systems the polarization difference can be uniquely computed. To demonstrate this, let us consider a simple one-dimensional model system with two point charges (A and B) as shown in Fig. 8a, where A carries a charge amount of +2*q* while B carries −*q* so that the system has a net charge. More complicated systems such as *solids* can be similarly handled by using the centers of Wannier functions. Now let A be displaced by a distance Δ_1_ and B by a distance Δ_2_ (Fig. 8b). The change in dipole moment (which is related to polarization by a volume factor) for the configuration (b) with respect to configuration (a), Δ*τ* = 2*q*Δ_1_ − *q*Δ_2_, is well defined, despite that the system is charged.

Furthermore, the change in polarization does not depend on whether an atom is to be fixed in the structural relaxation in real LDA calculations. For instance, let us fix the position of charge A as shown in Fig. 8c, which is equivalent to translating the whole system (including charges A, B, and the coordinate origin) from the configuration in Fig. 8b to the configuration in Fig. 8c. The origin needs to move from O to O′ since the dipole moment is a position operator which depends on the origin. Then, the change in the dipole moment for the configuration (c) with respect to the configuration (a) is Δ*τ*′ = 2*q*Δ_1_ − *q*Δ_2_, which is identical to Δ*τ*.

The above principle can be easily generalized to arbitrary systems. Regardless of which atom is to be fixed, the final structures after relaxation will have the same *relative* atomic geometry since the lengths and directions of chemical bonds are the same, except that the whole system is translationally (and rigidly) shifted. In the text, we have rigorously showed that the polarization *change* in charged systems is translationally invariant, and thus remains the same, no matter which atom is fixed in structural optimization.

## Additional Information

**How to cite this article**: Raeliarijaona, A. and Fu, H. Persistence of strong and switchable ferroelectricity despite vacancies. *Sci. Rep.* **7**, 41301; doi: 10.1038/srep41301 (2017).

**Publisher's note:** Springer Nature remains neutral with regard to jurisdictional claims in published maps and institutional affiliations.
